# Renditions on plastics tracking strategy: plastic-related small molecules as chemical fingerprints

**DOI:** 10.1590/1806-9282.20251138

**Published:** 2025-12-15

**Authors:** Ilker Sengul, Demet Sengul

**Affiliations:** 1 Giresun University, Faculty of Medicine, Division of Endocrine Surgery – Giresun, Turkey.; 2 Giresun University, Faculty of Medicine, Department of General Surgery – Giresun, Turkey.; 3 Giresun University, Faculty of Medicine, Department of Pathology – Giresun, Turkey.

We do pen these lines in order to commend, "Plastics tracking strategy: Plastic-related small molecules as chemical fingerprints," which proposes a novel and promising approach to address the complex challenge of tracking plastics throughout their life cycle^1^. The concept of using plastic-related organic small molecules, both endogenous and exogenous, as unique chemical fingerprints offers a powerful tool for understanding processes such as plastic processing, use, recycling, abandonment, and environmental aging pathology. As such, this strategy has the potential to significantly enhance our ability to manage plastics and their associated chemical burden. Whilst the proposed "chemical fingerprints tracking strategy" presents a compelling vision, we kindly wish to highlight several critical challenges that must be robustly addressed for its widespread and reliable implementation. Initially, the sheer complexity and unknown nature of chemicals within plastics present a formidable hurdle. The sources doth reveal that plastics are "multi-chemical cocktails," containing over 16,000 chemicals from intentional or unintentional additions, in addition to sorbed chemicals and environmental degradation products. The commentary explicitly states that "most chemicals and transformation products are unidentified," which directly hinders the development of a comprehensive chemical fingerprint tracking strategy. For a fingerprinting method to be truly effective as a "unique identity marker," a more complete characterization and understanding of this vast chemical landscape, including unknown additives, is urgently required. Second, the challenges in analytical and detection methodology for such diverse and complex small molecules are significant, which commentary rightly points out the necessity for novel analytical and detection methods that ensure comprehensive characterization with precision and cost-effectiveness, eliminate variations across different substrates, avoid interference from high concentrations of additives, and enable high-throughput analysis. Of note, without overcoming these technical analytical barriers, the practical application of tracking diverse chemical fingerprints across various environmental matrices remains challenging. Third, while multivariate statistical analysis tools, particularly machine learning algorithms, are proposed as future directions to handle large and complex datasets, their efficacy is contingent on the availability of "large and comprehensive datasets" for training. Furthermore, it is a substantial undertaking to generate such extensive, high-quality datasets for all possible plastic types, their endogenous and exogenous compounds, and their behavior under diverse environmental conditions. Moreover, the current limitations in understanding "unidentified plastic-related small molecules and their environmental behavior" further exacerbate this data gap, particularly for exogenous compounds crucial for tracking transport pathways and environmental aging. As a matter of fact, the authors have laid out a crucial framework for a chemical fingerprint tracking strategy that holds immense promise for plastic management and environmental health. However, the successful realization of this strategy hinges on significant investment in fundamental research to identify unknown chemicals, develop advanced analytical techniques, and build comprehensive, high-quality databases for robust machine learning applications and artificial intelligence^1^. Addressing these foundational challenges will be paramount to transforming this innovative concept into a practical and widely applicable tool for a truly transparent and traceable plastic life cycle. This issue merits further investigation. We thank Liu et al.^1^ for their study on plastic-related small molecules as chemical fingerprints^2,3^.

## Data Availability

The datasets generated and/or analyzed during the current study are available from the corresponding author upon reasonable request.
